# ZHX2 inhibits thyroid cancer metastasis through transcriptional inhibition of S100A14

**DOI:** 10.1186/s12935-022-02499-w

**Published:** 2022-02-12

**Authors:** Yankun Zhang, Min Sun, Lifen Gao, Xiaohong Liang, Chunhong Ma, Jinghui Lu, Xuetian Yue

**Affiliations:** 1 Key Laboratory for Experimental Teratology of Ministry of Education, Key Laboratory of Infection and Immunity of Shandong Province and Department of Immunology, School of Basic Medical Sciences, Cheeloo Medical College of Medicine, Shandong University, Jinan, 250012 China; 2 Department of Hernia and Abdominal Wall Surgery, General Surgery, Qilu Hospital, Cheeloo College of Medicine, Shandong University, Jinan, 250012 China; 3 Department of Cell Biology, School of Basic Medical Science, Cheeloo College of Medicine, Shandong University, Jinan, 250012 China

**Keywords:** ZHX2, Thyroid cancer, Metastasis, S100A14

## Abstract

**Background:**

Thyroid cancer is the most common malignant endocrine tumour, and metastasis has become the main reason for treatment failure. However, the underlying molecular mechanism of thyroid cancer metastasis remains poorly understood. We investigated the role of the tumour suppressor zinc fingers and homeoboxes 2 (ZHX2) in the metastasis of thyroid cancer.

**Methods:**

To study the role of ZHX2 in thyroid cancer metastasis, we evaluated the EMT process using cell migration, wound healing and lung metastatic tumour formation in vitro and in vivo models.

**Results:**

ZHX2 expression was significantly decreased in thyroid cancer tissues, which correlated with poor prognosis of thyroid cancer patients. ZHX2 knockdown significantly promoted the migration of thyroid cancer cells. Mechanistically, ZHX2 associated with the S100 calcium binding protein A14 (S100A14) promoter to decrease the transcription of S100A14. Moreover, S100A14 was highly expressed in human thyroid cancer samples, and its expression negatively correlated with ZHX2 expression.

**Conclusions:**

Inhibition of S100A14 attenuated the ZHX2 knockdown-induced enhanced metastasis of thyroid cancer cells both in vitro and in vivo. The evidence presented here suggests that ZHX2 inhibits the progression of thyroid cancer by blocking S100A14-mediated metastasis.

**Supplementary Information:**

The online version contains supplementary material available at 10.1186/s12935-022-02499-w.

## Background

Thyroid cancer is the most common malignant endocrine tumour [1]. The incidence of thyroid cancer has been steadily rising in recent years (an approximately 4% increase annually), and it represents the most rapidly increasing malignancy among all major cancers, tripling in the past 3 decades [2]. Most thyroid cancer cases originate in the follicular epithelium and can be divided into papillary thyroid cancer (PTC), follicular thyroid cancer (FTC) and anaplastic thyroid cancer (ATC) according to the pathologic type [3]. Patients diagnosed with thyroid cancer at an early stage have an excellent prognosis. However, individuals with large, invasive tumours and/or distant metastasis have a 5-year survival rate of only  ~ 40% [4, 5]. Thus, there is a need to better understand the molecular causes of thyroid cancer metastasis to develop new treatment options.

Zinc fingers and homeoboxes 2 (ZHX2) is a transcription factor belonging to the ZHX family that was initially identified based on its natural mutation in BALB/cJ mice [6, 7]. As a regulator of the oncofoetal genes alpha foetal protein (AFP) and glypican-3 (GPC3), ZHX2 inhibits the development of hepatocellular carcinoma [8–10]. ZHX2 is widely expressed and participates in many types of cancers, such as gastric cancer, lung cancer and clear cell renal cell carcinoma [11–13]. Recently, whole transcript microarray expression profiling detected the common expression of ZHX2 in brain metastatic PTC and primary brain tumours [14], suggesting that ZHX2 may play a role in thyroid cancer metastasis. However, whether and how ZHX2 is involved in the progression of thyroid cancer is unknown.

Metastasis is the major obstacle for thyroid cancer treatment. Mounting evidence has demonstrated that extracellular matrix (ECM) degradation plays an important role in cancer metastasis [15–17]. S100 proteins, a family of ECM proteins related to metastasis, are a group of multigene calcium-binding proteins [18]. Among them, S100A14 (S100 calcium binding protein A14), a member of the S100 family of proteins, has received more attention in tumour progression. S100A14 has been reported to be dysregulated in various types of tumours and involved in the proliferation, apoptosis and signal transduction of tumour cells [19]. In particular, S100A14 regulates the epithelial-mesenchymal transition (EMT) process to influence the metastasis of prostate cancer and cervical cancer [20, 21]. Further study clarified that S100A14 drives CCL2/CXCL5 signalling to promote breast cancer metastasis [22]. Until now, the role of S100A14 in thyroid cancer metastasis has not been elucidated.

To study the role of ZHX2 in thyroid cancer metastasis, we evaluated the EMT process using both in vitro and in vivo models. We found that overexpression of ZHX2 significantly restrained the thyroid cancer metastatic process, inhibiting wound healing and the migration of thyroid cancer cells. Mechanistically, ZHX2 inhibited S100A14 transcription. The expression of S100A14 negatively correlated with ZHX2 expression in human thyroid tumour specimens. More importantly, knockdown of S100A14 attenuated ZHX2 silencing-induced thyroid cancer cell migration both in vitro and in vivo. Therefore, targeting ZHX2 and S100A14 signalling may be a useful strategy to inhibit thyroid tumour progression.

## Materials and methods

### Patients and the clinical samples

Forty-nine thyroid tumour tissues and thirty-four para-tumour tissue samples were collected from Qilu Hospital, Shandong University (Additional file 1: Table S1). All of the patients had undergone surgery without preoperative radiation or chemotherapy before 2020.

### Cell lines and reagents

The human ATC cell lines 8305C (RRID: CVCL_1053), BHT101 (RRID: CVCL_1085) and KMH-2 (RRID: CVCL_S641) were kindly provided by the Stem Cell Bank, Chinese Academy of Sciences (Shanghai, China). The PTC cell line BHP10-3 (RRID: CVCL_6278) was obtained from Qilu Hospital of Shandong University [23]. All cell lines that were authenticated using STR profiling within the last 3 years were included. All experiments were performed with mycoplasma-free cells. They were maintained in Dulbecco’s modified Eagle’s medium (DMEM) (Thermo Fisher Scientific, C11995500CP) or minimum essential medium (MEM) (Thermo Fisher Scientific, 11095080) supplemented with 10% foetal bovine serum (FBS) (Biological Industries, 04-001-1A), 100 U/mL penicillin, 100 mg/mL streptomycin (Solarbio, P1400) and 2 mM L-glutamine (Solarbio, G0200) and incubated at 37 °C in an incubator with 5% CO_2_ and saturated humidity. The ZHX2-expressing vector pcDNA3-ZHX2-HA has been described previously [24]. Polymerase chain reaction (PCR)-amplified human S100A14 was cloned into the pcDNA3.0 vector. The primers used for cloning are shown in Additional file 2: Table S2. The siRNAs against ZHX2 and S100A14 (Additional file 2: Table S2) were synthesized by the GenePharma Inc. (Shanghai, China). These siRNAs were transfected into cells using the Lipofectamine™ 2000 Transfection Reagent (Invitrogen, 11668019). After incubatation for 48 h, the cells were collected for RNA or protein extraction.

### Quantitative real-time PCR (RT-qPCR)

Total RNA of cells and tissues was extracted using TRIzol reagent (TIANGEN Biotech, DP424) and reverse transcribed into cDNA with a Revert Aid First Strand cDNA Synthesis Kit (Thermo Fisher Scientific, K1622). Quantitative PCR (qPCR) was carried out using a BioRad C1000 Thermal Cycler CFX96 Real-Time System with ChamQ Universal SYBR qPCR Master Mix (Vazyme Biotech, Q711). The primers used in this article are shown in Additional file 2: Table S2.

### Western blotting

Thyroid tissues were homogenized in cell lysis buffer (Beyotime, P0013), and protein extracts were quantified by BCA protein assays (Beyotime, P0009). Equal amounts of proteins were loaded in SDS–polyacrylamide electrophoresis gels, transferred to Immobilon-P membranes (Millipore, Billerica) and incubated overnight at 4 °C with the following primary antibodies: mouse anti-GAPDH (Proteintech, 60004, 1:5,000), rabbit anti-ZHX2 (Proteintech, 20136-1-AP, 1:2,000), rabbit anti-S100A14 (Proteintech, 10489-1-AP, 1:500), rabbit anti-N-cadherin (Proteintech, 22018-1-AP, 1:2000), rabbit anti-E-cadherin (Proteintech, 20874-1-AP, 1:5000) rabbit anti-vimentin (Proteintech, 10366-1-AP, 1:2000), and mouse anti-beta actin (Proteintech, 66009-1-Ig, 1:5000). The membranes were washed with PBST 3 times and subsequently incubated with secondary HRP-conjugated anti-mouse (Proteintech, SA00001-1, 1:5000) or anti-rabbit IgG secondary antibodies (Proteintech, SA00001-2, 1:5000). The signal was detected by enhanced chemiluminescence (ECL) reagent (Millipore, WBULS0500) using the Tanon Bio-Imaging Systems (Tanon, 4600).

### Immunohistochemical staining assay

Thyroid cancer tissues were fixed in 4% formaldehyde solution to make paraffin sections. The deparaffinized sections were permeabilized with 0.3% Triton X-100 and blocked with 5% bovine serum albumin (Solarbio, C1032) following incubation with antibodies against ZHX2 (Proteintech, 20136-1-AP) and S100A14 (Proteintech, 10489-1-AP) overnight at 4 °C. The slips were then incubated with secondary antibodies and detected by DAB staining. Images were captured by microscopy (Olympus, Japan). Each section was counted independently by three pathologists and scored according to the staining intensity (no staining  = 0; weak staining  = 1; moderate staining  = 2; strong staining  = 3) and the number of stained cells (0–5% = 0; 6–25% = 1; 26–50% = 2; 51–75% = 3; 76–100% = 4) [25]. The final score was calculated using the following formula: intensity × stained number.

### Migration assay

Briefly, 2 × 10^4^ thyroid cancer cells with or without ZHX2 overexpression in 200 μL of serum-free medium were added to the upper chambers of the Transwell plate, and the lower chambers were filled with 800 μL of medium containing 10% FBS as a chemoattractant. After 24 h, the migrated cells were fixed in 4% paraformaldehyde and stained with 1% crystal violet, and the nonmigratory cells on the upper surface of the chambers were removed by a cotton swab. Then, at least four random fields were selected and observed by microscopy (Olympus, Japan).

### Wound-healing assays

Wound healing assays were performed in 6-well plates with confluent cells, and scratches were generated using 200 μL pipette tips. The wells were then washed three times with FBS-free medium and cultured for an additional 24 h, followed by the assessment of the relative wound closure areas using microscopy (Olympus, Japan).

### Luciferase reporter assay

The S100A14 gene promoter region was cloned into the pGL3-basic vector. The firefly luciferase reporter pGL3-S100A14 vector and Renilla luciferase plasmid (pRL-TK) were transiently co-transfected into the cells transfected with the pcDNA3-ZHX2 or control vector. pRL-TK was used for normalizing the transfection efficiency. Luciferase activities were measured by using a dual luciferase reporter assay kit (E1960, Promega). Primers for plasmid construction are shown in Additional file 2: Table S2.

### chromatin-immunoprecipitation (ChIP) assay

ZHX2 overexpression vector (pcDNA3-ZHX2-HA)-transfected 8305C cells were harvested for the ChIP assay by using the EZ-Magna ChIP™ A/G Chromatin Immunoprecipitation Kit (Millipore, 17-10086). Briefly, cells were fixed and sonicated to shear DNA to 200–1000 bp. The protein-DNA complexes were immunoprecipitated with anti-HA antibody (Proteintech, 51064-2-AP) or control IgG. The DNA fragments were collected and purified according to the manufacturer’s protocol. qPCR was performed using the primers shown in Additional file 2: Table S2.

### Animal experiments

BALB/c nude mice (6–7 weeks of age) were provided by Charles River and maintained under specific pathogen-free (SPF) conditions. ZHX2 knockdown or control 8305C cells were transduced with viral supernatants containing a recombinant lentivirus vector LV-shNC or LV-shS100A14, respectively. Lentiviruses expressing shRNAs against ZHX2 and shS100A14 were purchased from Shanghai GenePharma. The cells were collected, and the knockdown efficiency was verified by western blotting. Then, 8305C cells (1 × 10^6^) with ZHX2 and S100A14 knockdown individually or simultaneously were injected into BALB/c nude mice via the tail vein. On the 60th day of the experiment, mice were sacrificed, the total lung was weighed and lung tissues were collected for haematoxylin and eosin (H&E) assays. Randomly selected fields of H&E-stained lung tissues were captured. Images were captured by microscopy (Olympus, Japan). The number of migrated clones and migrated areas were calculated using ImageJ.

All animals were killed by the inhalation of carbon dioxide. Putting the animals into a closed transparent container, carbon dioxide was injected into the container at a rate of 20% per minute until the animals died.

### Statistical analysis

Statistical analysis was carried out with GraphPad Prism 8 software. Data are presented as the mean  ±  standard deviation (SD). One-way ANOVA or Student’s *t* test was used to assess statistical significance. Statistical significance was reported as highly significant using *(*p*  < 0.05), **(*p*  < 0.01) or ***(*p*  < 0.001).

## Results

### Low expression of ZHX2 correlates with poor prognosis in thyroid cancer

**Fig. 1:**
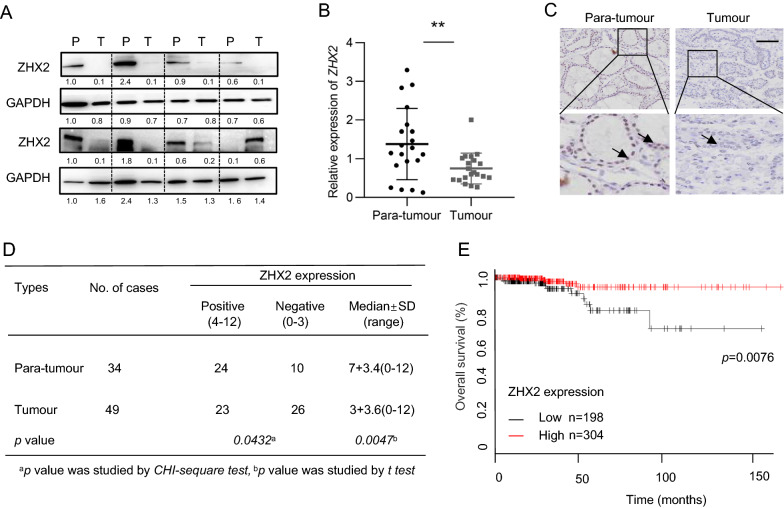
Low expression of ZHX2 was associated with poor prognosis in thyroid cancer. **A** ZHX2 expression was measured in human tissue samples using western blotting (WB). GAPDH was used as the internal control. Thyroid tumour tissues (T); adjacent non-tumour tissues (P). **B** *ZHX2* mRNA levels were evaluated by RT–qPCR in human specimens. **p < 0.01, the *p value* was determined by Student’s *t *test. **C** Representative images of ZHX2 immunostaining in thyroid adjacent non-tumour and thyroid tumour tissues (Image magnifications of 100 × , bar: 100 μm). **D** Statistical analysis of ZHX2 expression in thyroid adjacent non-tumour and thyroid tumour tissues. Significant differences were determined by the chi-squared test. **E** Kaplan–Meier survival curves of thyroid cancer patients with ZHX2 expression were analysed by online software

To determine the protein levels of ZHX2 in thyroid tumours, we employed western blotting for collected thyroid tumour samples. As shown in Fig. 1A, adjacent non-tumour tissues (referred to as P) showed higher levels of ZHX2 protein than thyroid cancer tissues (referred to as T). Consistently, significantly lower expression of *ZHX2* mRNA in tumours was confirmed by RT-qPCR analysis (Fig. 1B). To further evaluate the location and expression of ZHX2 protein on thyroid cancer cells, we detected ZHX2 protein in thyroid cancer tissues using immunohistochemistry (IHC). We observed ZHX2-positive staining (arrow) in the nuclei of adjacent normal cells and ZHX2-negative or weak ZHX2-positive staining in tumour cells, as exemplified in Fig. 1C. Further semi-quantitative scoring showed that ZHX2 protein levels were significantly higher in adjacent normal tissues than in tumour tissues (Fig. 1D). The results of the Kaplan–Meier curve (http://kmplot.com) revealed that low expression of ZHX2 correlated with poor prognosis of thyroid cancer patients (Fig. 1E). Together, these results show that ZHX2 expression is decreased in thyroid cancer.

### ZHX2 inhibits the migration of thyroid cancer in vitro

**Fig. 2:**
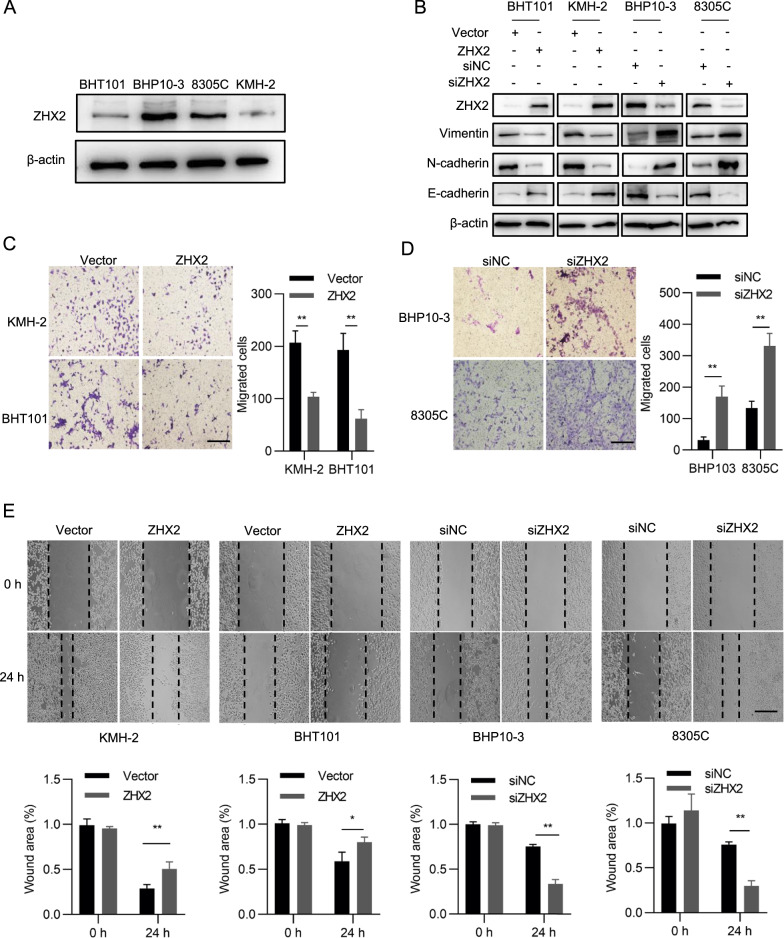
ZHX2 restrains the migration of thyroid cancer cells. **A** The protein levels of ZHX2 in BHT101, BHP10-3, 8305C and KMH-2 cells were examined by WB. **B** EMT-related proteins N-cadherin, E-cadherin and vimentin were assayed in ZHX2-overexpressing KMH-2 and BHT101 cells or ZHX2-knockdown BHP10-3 and 8305C cells by WB. Thyroid cancer cells with the indicated manipulation were used to determine the migratory ability by using Transwell (**C**, **D**) and wound healing assays (**E**). The migrated cells were photographed. Image magnifications: 100 ×  (**C**, **D**), 40 ×  (**E**). Bar: 200 μm (**C**, **D**), 500 μm (**E**). Representative pictures are shown. Data are presented as the mean ± SD. Student’s *t *test, *p  < 0.05, **p  < 0.01

To further investigate the role of ZHX2 in thyroid cancer metastasis, we measured the migration ability of thyroid tumour cell lines with modulated levels of ZHX2. First, ZHX2 protein levels were detected in BHP10-3, BHT101, 8305C and KMH-2 cell lines. As shown in Fig. 2A, ZHX2 protein levels were lower in BHT101 and KMH-2 than in BHP10-3 and 8305C cell lines. Therefore, we overexpressed ZHX2 in BHT101 and KMH-2 cells and knocked down ZHX2 in BHP10-3 and 8305C cells to evaluate the migratory ability. Vimentin, N-cadherin and E-cadherin are well-known markers that indicate cell migration [26]. Here, we observed that ZHX2 protein levels negatively correlated with the protein levels of vimentin and N-cadherin, and positively correlated with the protein levels of E-cadherin (Fig. 2B), indicating that ZHX2 restrains the migration of thyroid cancer cells. To verify this hypothesis, Transwell assays were performed. As displayed in Fig. 2C, the migrated cell numbers were lower in ZHX2-overexpressing KMH-2 and BHT101 cells than in control cells. Knockdown of ZHX2 by siRNAs increased the migration ability of both BHP10-3 and 8305C cells (Fig. 2D). Further wound healing assays also showed that cell motility was reduced in ZHX2-overexpressing thyroid cancer cell lines and increased in ZHX2 knockdown thyroid cancer cell lines (Fig. 2E). Above all, the data demonstrate that ZHX2 restrains the migration of thyroid cancer cells.

### ZHX2 negatively correlates with S100A14 in thyroid cancer

**Fig. 3:**
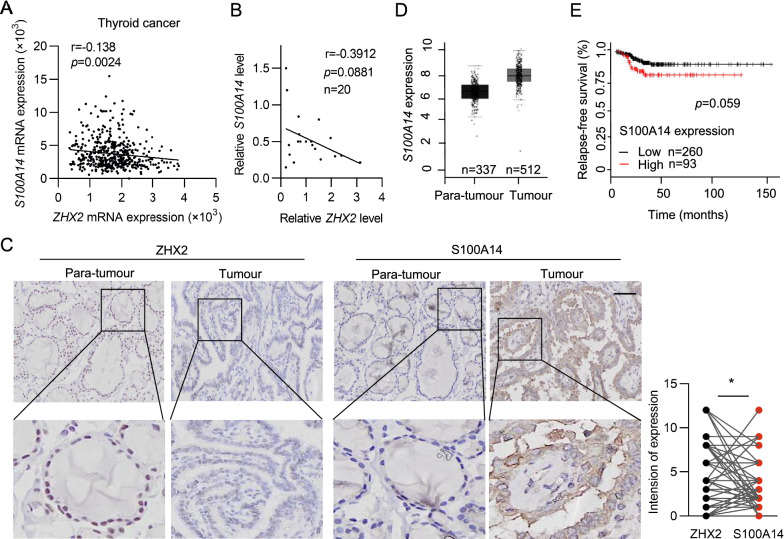
ZHX2 inhibits S100A14 expression in thyroid cancer. **A** Correlations between *ZHX2* and *S100A14* mRNAs were analysed using the cBioPortal thyroid cancer dataset. **B** RT–qPCR analysis was used to determine the relationship between *ZHX2* and *S100A14* mRNAs in human specimens. **C** ZHX2 and S100A14 immunostaining was performed in thyroid para-tumour tissues and thyroid tumour tissues. Representative images are displayed (Image magnifications of 100 × , bar: 100 μm, left). Statistical analysis of ZHX2 and S100A14 correlation in samples (right). **D** *S100A14* expression was determined in thyroid para-tumour tissues and thyroid tumour tissues from GEPIA datasets. **E** Kaplan–Meier relapse-free survival of thyroid cancer patients with S100A14 is presented. Data are presented as the mean ± SD. Student’s *t *test, *p < 0.05. **A**, **B** Pearson’s correlation coefficients (r) and *p* values (*p*) for two-sided correlation tests are shown

ECM degradation is an important biological process of metastasis [15–17]. The S100 protein family, belonging to ECM proteins, is closely related to tissue repair and tumour progression and has drawn much clinical attention in recent years [27–29]. Based on these findings, we evaluated the functional interaction between ZHX2 and S100 family members. Interestingly, through analysis of publicly available cBioPortal data, we detected a negative correlation of ZHX2 with multiple members of S100 (Additional file 3: Table S3). Among them, S100A14, on the top of the list, showed a significant negative correlation with ZHX2 expression (Fig. 3A). Consistent with publicly available data, a negative correlation between ZHX2 and S100A14 mRNA expression (Pearson’s r  = − 0.3912) was also presented in our clinical samples (Fig. 3B). Furthermore, we also observed that the protein level of ZHX2 was negatively correlated with the S100A14 protein level in both thyroid cancer tissues and adjacent non-tumour tissues, as exemplified in Fig. 3C. These results indicate that ZHX2 inhibits the expression of S100A14 in thyroid cancer. Then, we aimed to determine the role of S100A14 in thyroid cancer. Notably, it was found that thyroid cancer tissues had elevated S100A14 mRNA levels compared with adjacent non-tumour tissues by analysing GEPIA (http://gepia.cancer-pku.cn) (Fig. 3D). More importantly, Kaplan–Meier curves revealed that high expression of S100A14 correlated with poor prognosis in thyroid cancer patients (Fig. 3E), suggesting that S100A14 is an oncogene of thyroid cancer. Therefore, these data show that ZHX2 might inhibit thyroid cancer via S100A14.

### ZHX2 binds to the S100A14 promoter to repress its transcription

**Fig. 4:**
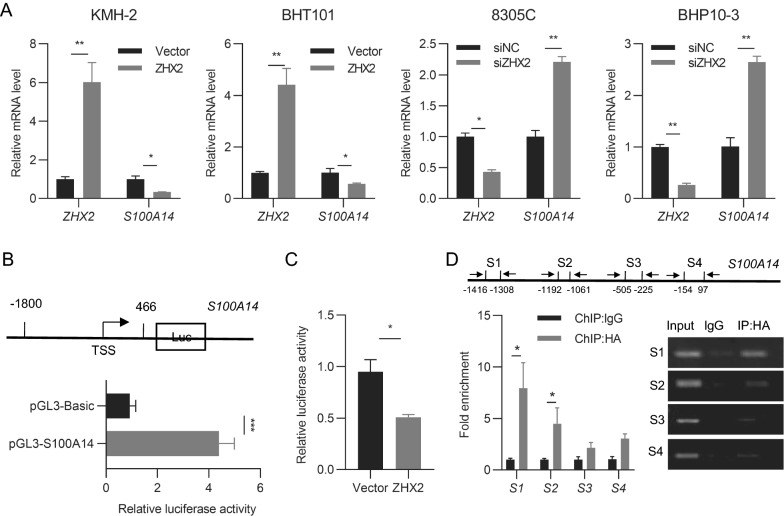
ZHX2 binds to the S100A14 promoter to repress its expression. **A** RT–qPCR analysis of ZHX2 and S100A14 mRNA levels in thyroid cancer cells with ZHX2 overexpression or knockdown. **B** Diagram of the S100A14 promoter region and S100A14 promoter luciferase constructs are presented in the top panel. The promoter activity of pGL3-S100A14 was verified by the dual luciferase assay (lower panel). **C** S100A14 promoter activity was measured in 8305C cells with or without ZHX2 overexpression using a dual fluorescent reporter assay. **D** The ChIP assay was used to analyse ZHX2 occupancy on the promoter of S100A14. 8305C cells were transfected with a plasmid encoding ZHX2. HA antibody was used to pull down DNA–protein complexes in these cells. Primers targeting S100A14 promoter regions (S1–S4) are shown in the top panel. The lower panel presents the quantification of qPCR (left) and semiquantitative images (right). Data are presented as the mean ± SD. Student’s *t *test. *p  < 0.05, **p  < 0.01, ***p  < 0.001

Since ZHX2 is a well-known transcription factor, we examined *S100A14* mRNA levels in thyroid cancer cell lines with ZHX2 manipulation. ZHX2 overexpression vector or siRNAs were transfected into thyroid cancer cell lines. RT–qPCR analysis showed that *S100A14* mRNA levels were dramatically decreased in ZHX2-overexpressing KMH-2 and BHT101 cells. Reciprocally, knockdown of ZHX2 obviously elevated the mRNA level of *S100A14* in 8305C and BHP10-3 cells (Fig. 4A), indicating that ZHX2 might repress S100A14 expression at the transcriptional level. To verify this hypothesis, we performed dual luciferase and chromatin immunoprecipitation (ChIP) assays. We successfully cloned the S100A14 promoter region (− 1800 to  + 466 bp) in the pGL3-basic plasmid (pGL3-S100A14) and verified its promoter activity (Fig. 4B). The results of cotransfection and dual-luciferase assays showed that enforced expression of ZHX2 greatly inhibited S100A14 promoter activity in 8305C cells (Fig. 4C). Furthermore, a ChIP assay was performed in pcDNA3-ZHX2-HA-transfected 8305C cells to analyse the occupancy of ZHX2 in the promoter of S100A14. Bioinformative analysis identified four putative ZHX2 binding motifs on the S100A14 promoter by the Eukaryotic Promoter Database EPD (http://epd.vital-it.ch) [30, 31]. As shown in Fig. 4D, the locations of four putative ZHX2 binding sites on the promoter region of S100A14 are presented. Among them, section 1 (− 1416 to − 1308 bp) is the major ZHX2 binding site, as section 1 primers induced significantly increased fold enrichment in the qPCR assay and an obviously amplified band in the semiquantitative PCR assay. Taken together, these data show that ZHX2 transcriptionally suppresses the expression of S100A14.

### ZHX2 restrains thyroid cancer cell migration via repression of S100A14 in vitro

**Fig. 5:**
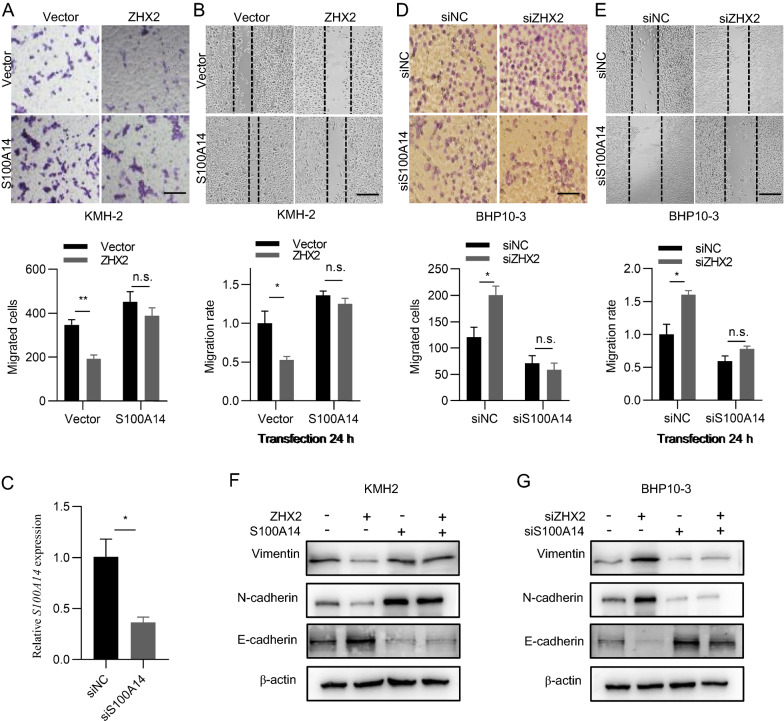
ZHX2 restrains thyroid cancer cell migration via repression of S100A14 in vitro. **A**–**F** ZHX2-overexpressing or ZHX2-knockdown KMH-2 and BHP10-3 cells were transfected with S100A14 or siS100A14, respectively. Transwell assays (**A**, **D**), wound healing assays (**B**, **E**) and protein levels of EMT markers N-cadherin, E-cadherin and vimentin (**F**, **G**) were assessed in these cells. The migrated cells were photographed. Image magnifications: 200 ×  (**A**, **D**), 40 ×  (**B**, **E**). Bar: 100 μm (**A**, **D**), 500 μm (**B**, **E**). **C** RT-qPCR analysis for knockdown of S100A14 in BHP10-3 cells were shown. Data are the mean ± SD. One-way ANOVA. *p < 0.05, **p < 0.01, *ns* not significant

To verify whether the inhibition of thyroid cancer migration by ZHX2 was achieved by repressing S100A14, we performed the following experiments. As shown in Fig. 5A, overexpression of ZHX2 inhibited cell migration, and ectopic expression of S100A14 promoted cell migration. More importantly, ectopic expression of S100A14 significantly abolished the ZHX2-mediated inhibitory effect on cell migration in KMH-2 cells. Consistently, ectopic expression of S100A14 eliminated the ZHX2-induced inhibition of wound healing in KMH-2 cells (Fig. 5B). To verify these results, we performed Transwell and wound healing assays using BHP10-3 cells with ZHX2 and/or S100A14 knockdown, simultaneously or independently. The efficiency of S100A14 knockdown in BHP10-3 cells was verified by RT-qPCR (Fig. 5C). Cell migration and the wound healing ability were increased in ZHX2 knockdown BHP10-3 cells. Notably, this inhibition was reversed when siS100A14 was cotransfected with siZHX2 (Fig. 5D, E). In accordance, western blot analysis revealed that ZHX2 protein levels were negatively associated with the protein levels of vimentin and N-cadherin, positively correlated with E-cadherin. S100A14 protein levels were positively associated with vimentin and N-cadherin proteins, negatively correlated with E-cadherin. Cotransfection of S100A14 with ZHX2 eliminated the ectopic expression of ZHX2-induced decreases in vimentin and N-cadherin proteins, and increases in E-cadherin proteins in KMH-2 cells (Fig. 5F). Reciprocally, silencing S100A14 abolished the accumulation of vimentin and N-cadherin proteins in ZHX2 siRNA-transfected BHP10-3 cells (Fig. 5G). Above all, ZHX2 inhibits thyroid cancer migration via S100A14.

### ZHX2 inhibits lung metastatic tumour formation of thyroid cancer cells via S100A14

**Fig. 6:**
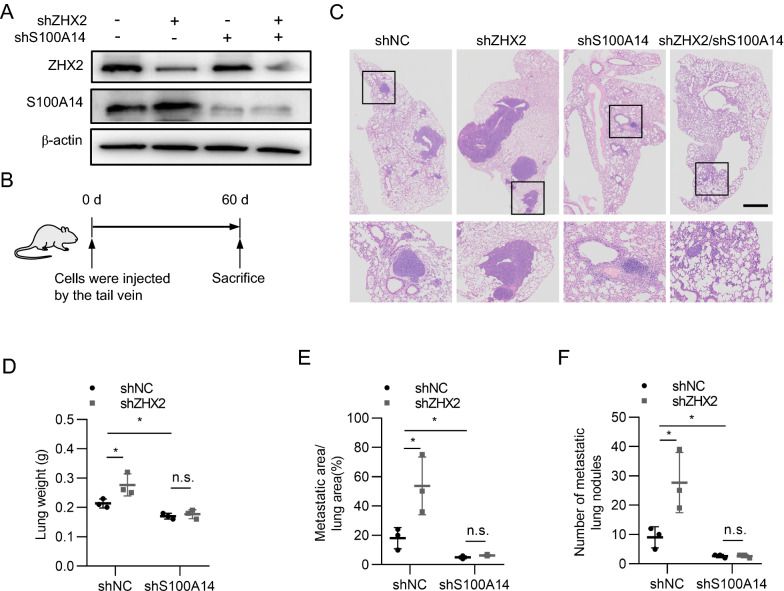
ZHX2 inhibits thyroid cancer cell lung metastasis through S100A14 in vivo. Thyroid cancer cells (8305C) with or without ZHX2 knockdown were transduced with shS100A14 or their control viruses. **A** The knockdown efficiency of shZHX2 or shS100A14 was determined by WB. These cells were injected into nude mice via the tail vein to establish lung metastasis models. **B** The diagram shows the flow of the experiment. **C** Mouse lung tissues were collected to perform H&E staining. Representative images of each group are presented. (Image magnifications of 20 × , scale bar: 500 μm). Quantification of the lung weight (**D**), metastatic lung fraction (**E**) and number of metastatic lung nodules (**F**) was performed. Data are the mean ± SD. One-way ANOVA. *p  < 0.05, *ns* not significant

To further confirm that ZHX2 restrains thyroid cancer metastasis via S100A14, we performed lung metastasis assays in mouse models. Stable cell lines with varied ZHX2 and S100A14 levels were established through transduction of 8305C with ZHX2-shRNA and/or S100A14-shRNA viruses, respectively. As shown in Fig. 6A, endogenous ZHX2 or S100A14 was efficiently knocked down by ZHX2-shRNA or S100A14-shRNA, respectively. Then, four groups of cells (shNC, shZHX2, shS100A14 and shZHX2/shS100A14) were injected into nude mice via the tail vein to evaluate the formation of lung metastatic tumours (Fig. 6B). The results showed that ZHX2 knockdown increased the lung weight and promoted the formation of lung metastatic tumours, displayed as heavier lungs, larger migrated areas and more metastatic tumours in the shZHX2 group than in the shNC group. Knockdown of S100A14 markedly decreased the lung weight, migrated area and number of metastatic tumours compared to those of the shNC-transduced group. More importantly, S100A14 knockdown abolished the ZHX2 silencing-induced enhancement of lung weight and lung metastasis (Fig. 6C–F). Therefore, these data demonstrate that ZHX2 inhibits thyroid cancer metastasis via S100A14 in in vivo mouse models.

## Discussion

As an endocrine malignancy, thyroid cancer is commonly seen in the clinic [32]. Although thyroid cancers such as PTC have a favourable prognosis and a low death rate, a certain number of patients develop more aggressive forms that are unresponsive to radioactive iodine and chemotherapy, resulting in increased incurability and patient morbidity and mortality [33]. For these reasons, the identification of key molecules responsible for thyroid cancer metastasis is urgent and highly demanded for improving the clinical outcome. In this study, we found that low expression of ZHX2 led to upregulation of S100A14, which in turn promoted cell migration, wound healing and lung metastatic tumour formation. These results suggest that ZHX2 is a key regulator of thyroid cancer metastasis, which transcriptionally represses S100A14 expression to inhibit thyroid cancer metastasis. Therefore, strategies aiming to manipulate ZHX2 and S100A14 might be helpful to treat thyroid cancer.

Identified as a transcription factor, ZHX2 inhibits the development of hepatocellular carcinoma (HCC). Previous reports, including ours, showed that ZHX2 represses HCC oncogenes, such as AFP, GPC3, cyclin A/cyclin E and MDR1, which are involved in many biological processes of HCC development, including cell proliferation, cell migration and chemoresistance [7, 8, 10, 34]. However, the role of ZHX2 in thyroid cancer has not been reported. Here, we found that low expression of ZHX2 was associated with poor prognosis in patients with thyroid tumours. Further study showed that silencing ZHX2 increased cell migration and wound healing in thyroid cancer cell lines. In line with our findings, overexpression of ZHX2 reduced the migratory ability of lung cancer cells via the p38MAPK signalling pathway [11]. However, a report showed that ZHX2 promotes the migration of clear cell renal carcinoma cells [35]. Therefore, ZHX2 might play different roles in tumour metastasis depending on the tumour type. Previous study reported that ZHX2 is widely expressed in many tissues, such as liver, lung, kidney and brain [36]. Thus, understanding the role of ZHX2 in different types of cancers will be beneficial for cancer-targeting therapy.

One of the important questions is how ZHX2 inhibits the metastasis of thyroid cancer. Based on bioinformatics analysis, we found that ZHX2 negatively correlated with S100A14 in thyroid cancer. As a member of the S100 family, S100A14 (also known as breast cancer membrane protein 84) is a newly identified member of the S100 protein family and has recently gained significant attention in cancer research [37]. Mounting evidence has shown that S100A14 is involved in many biological processes of cancer development, including cell proliferation, apoptosis, cell migration and cell differentiation [19, 38–40]. In particular, it is involved in the EMT processes of cell migration and invasion, which are associated with the outcome of thyroid cancer [41, 42]. S100A14 belongs to extracellular matrix proteins that are involved in the biological process of EMT [20, 43]. Here, we found that knockdown of S100A14 abolished ZHX2 silencing-induced cell migration both in vitro and in vivo, demonstrating that ZHX2 is involved in thyroid cancer metastasis through S100A14. More importantly, our data illustrated that ZHX2 bound to the S100A14 promoter to repress its transcription. Consistent with our findings, many reports have shown that ZHX2 inhibits the promoter activity of many oncogenes, such as AFP, GPC3, MDR1 and Cyclin A/E [8–10, 34]. Further studies showed that ZHX2 binds to specific DNA sequences through interaction with NF-YA or NF-κB, depending on the tissue context [13, 44]. Here, ChIP assays demonstrated that ZHX2 could pull down DNA fragments in the S100A14 promoter region. Whether ZHX2 directly or indirectly interacts with these DNA fragments still needs to be elucidated. In addition, the NF-κB signalling pathway participates in PTC progression by regulating the EMT process [45]. It is possible that ZHX2 regulates thyroid cancer metastasis through multiple pathways. Nevertheless, our data demonstrated that ZHX2 inhibits thyroid cancer metastasis by repressing S100A14 expression at the transcriptional level.

In conclusion, we identified S100A14 as a new target of ZHX2 and revealed that ZHX2 bound to the S100A14 promoter to repress its transcription, which in turn inhibited thyroid cancer metastasis. Our findings expand the understanding of the molecular mechanism underlying thyroid cancer metastasis and provide a potentially effective target for thyroid cancer diagnosis and therapy.

## Supplementary Information

**Additional file 1: Table S1. **The information of patients involved in this study.

**Additional file 2: Table S2.** Synthetic oligonucleotides.

**Additional file 3: Table S3.** The cBioPortal analysis negative correlation of ZHX2 expression with S100 family in thyroid cancer patients [Thyroid Carcinoma (TCGA, PanCancer Atlas)] listed in the table.

## Data Availability

The data that supports the findings of our study are available from the corresponding author upon reasonable request.

## Abbreviations

ZHX2: Zinc fingers homeoboxes
PTC: Papillary thyroid cancer
FTC: Follicular thyroid cancer
ATC: Anaplastic thyroid cancer
S100A14: S100 calcium binding protein A14
RT-qPCR: Quantitative real-time PCR
qPCR: Quantitative PCR
ChIP: Chromatin immunoprecipitation
IHC: Immunohistochemical
EMT: Epithelial mesenchymal transition
