# Supplementary material for: ZHX2 inhibits thyroid cancer metastasis through transcriptional inhibition of S100A14
Source: Cancer Cell Int. 2022 Feb 12;22:76. doi: 10.1186/s12935-022-02499-w (PMC8840030; doi:10.1186/s12935-022-02499-w)
Supplement: Supplementary file 2 — Additional file 2: Table S2. Synthetic oligonucleotides. [file 12935_2022_2499_MOESM2_ESM.docx]

**Table S2. Synthetic Oligonucleotides.**

| **Primer for RT-PCR** | |
| --- | --- |
| **symbol** | **sequence** |
| h-ZHX2-qF | GGTTCGGACATCACAAGTAGTAG |
| h-ZHX2-qR | GGTGTGCCGATTCCTTTCTCT |
| h-β-actin-qF | AGTTGCGTTACACCCTTTC |
| h-β-actin-qR | CCTTCACCGTTCCAGTTT |
| h- S100a14-F | GGTTGGCAATTTTCTCTTCCAGG |
| h- S100a14-R | TCTGTCAATGCCCAAGCACC |
| **Primer for ChIP-PCR** | |
| **symbol** | **sequence** |
| S1-ChIP-F | GGCTGTCACTGCCTCACTG |
| S1-ChIP-R | AGTCTGGGAGTCTCTCTGAGGTC |
| S2-ChIP-F | CCTGAGCCTCCTCCTGAGG |
| S2-ChIP-R | CTCAATGGAGAGGACCTTCCAACAAT |
| S3-ChIP-F | CAGGCAGGCTTGAGTGTCAGTATC |
| S3-ChIP-R | GTCACAGAGAAAGAGAAGCGAGAAGG |
| S4-ChIP-F | TCATGGTAGGCTCTGGAACTCTGG |
| S4-ChIP-R | TTACCCTAAGTGTGCCCTCTGGAC |
| **Plasmid****’s construction** |  |
| **symbol** | **sequence** |
| pcDNA3-S100A14-HA-F | CGGGGTACCATGGGACAGTGTCGGTCAGCCA |
| pcDNA3-S100A14-HA-R | CCGCTCGAGCTAAGCGTAGTCTGGTACGTCGTAAGGGTAGTGCCCCCGGACAGGCCT |
| pGL3-S100A14-F | CGGGGTACCGTCTTAGTTAATTCTCACAGTC |
| pGL3-S100A14-R | CTAGCTAGCGGTGCTCACTGTGTCTGGTC |
| **Synthetic interfering RNA** | |
| **symbol** | **sequence** |
| Srcamble-sense | UUCUCCGAACGUGUCACGUTT |
| Srcamble-antisense | ACGUGACACGUUCGGAGAATT |
| homo-ZHX2-1-sense | GCAGAACUGGAUCGGCUAATT |
| homo-ZHX2-1-antisense | UUAGCCGAUCCAGUUCUGCTT |
| homo-S100A14-sense | GAGUUUCUGGGAGGCUGAUUTT |
| homo-S100A14-antisense | AAUCAGCUCCCAGAAACUCUTT |
