# Supplementary material for: ZHX2 inhibits thyroid cancer metastasis through transcriptional inhibition of S100A14
Source: Cancer Cell Int. 2022 Feb 12;22:76. doi: 10.1186/s12935-022-02499-w (PMC8840030; doi:10.1186/s12935-022-02499-w)
Supplement: Supplementary file 3 — Additional file 3: Table S3. The cBioPortal analysis negative correlation of ZHX2 expression with S100 family in thyroid cancer patients [Thyroid Carcinoma (TCGA, PanCancer Atlas)] listed in the table. [file 12935_2022_2499_MOESM3_ESM.docx]

**Table S3. The cBioPortal analysis negative correlation of ZHX2 expression with S100 family in thyroid cancer patients (Thyroid Carcinoma (TCGA, PanCancer Atlas)) listed in the table**

| Correlated gene | Spearman’ correlation | p-Value |
| --- | --- | --- |
| S100A14 | -0.137 | 2.66e-3 |
| S100G | -0.096 | 1.295e-5 |
| S100A12 | -0.0319 | 6.077e-5 |
| S100A1 | -0.0288 | 8.079e-5 |
| S100A7A | -0.0103 | 9.472e-5 |
